# Multiparametric in vitro and in vivo analysis of the safety profile of self-assembling peptides

**DOI:** 10.1038/s41598-024-54051-7

**Published:** 2024-02-22

**Authors:** Ariel Ramirez-Labrada, Llipsy Santiago, Cecilia Pesini, Marta Arrieta, Maykel Arias, Adanays Calvo Pérez, Maria Gessica Ciulla, Mahdi Forouharshad, Julian Pardo, Eva M. Gálvez, Fabrizio Gelain

**Affiliations:** 1 Immunotherapy, Cytotoxicity, Inflammation and Cancer, Aragón Health Research Institute (IIS Aragón), Biomedical Research Centre of Aragón (CIBA), Zaragoza, Spain; 2 Nanotoxicology and Immunotoxicology Unit (UNATI), Aragón Health Research Institute (IIS Aragón), Biomedical Research Centre of Aragón (CIBA), Zaragoza, Spain; 3 Center for Biomedical Research in the Network of Infectious Diseases (CIBERINFEC), Carlos III Health Institute, Zaragoza, Spain; 4 Instituto de Carboquimica (ICB), CSIC, Zaragoza, Spain; 5 WorldPathol Global United S.A. (WGUSA), Zaragoza, Spain; 6 Department of Microbiology, Preventive Medicine and Public Health, University of Zaragoza, Zaragoza, Spain; 7 Center for Nanomedicine and Tissue Engineering (CNTE), ASST Grande Ospedale Metropolitano Niguarda, 20162 Milan, Italy; 8 Tissue Engineering Unit-ISBREMIT-IRCCS Casa Sollievo Della Sofferenza, Via Cappuccini 1, 71013 San Giovanni Rotondo, FG Italy

**Keywords:** Neuroscience, Neurology, Nanoscience and technology

## Abstract

Self-assembling peptides (SAPs) have gained significant attention in biomedicine because of their unique properties and ability to undergo molecular self-assembly driven by non-covalent interactions. By manipulating their composition and structure, SAPs can form well-ordered nanostructures with enhanced selectivity, stability and biocompatibility. SAPs offer advantages such as high chemical and biological diversity and the potential for functionalization. However, studies concerning its potentially toxic effects are very scarce, a limitation that compromises its potential translation to humans. This study investigates the potentially toxic effects of six different SAP formulations composed of natural amino acids designed for nervous tissue engineering and amenable to ready cross-linking boosting their biomechanical properties. All methods were performed in accordance with the relevant guidelines and regulations. A wound-healing assay was performed to evaluate how SAPs modify cell migration. The results in vitro demonstrated that SAPs did not induce genotoxicity neither skin sensitization. In vivo, SAPs were well-tolerated without any signs of acute systemic toxicity. Interestingly, SAPs were found to promote the migration of endothelial, macrophage, fibroblast, and neuronal-like cells in vitro, supporting a high potential for tissue regeneration. These findings contribute to the development and translation of SAP-based biomaterials for biomedical applications.

## Introduction

Biomaterials are under widespread investigation for experimental and therapeutic applications. Although various classes of biomaterials have been used, synthetic or naturally derived polymers are the predominant choice for biomedical applications mainly due to low toxicity and good biocompatibility. One of them, the SAPs, is arousing great attention as novel biomaterials in different fields, such as nanomedicine, nanomaterials, and nanobiotechnology, thanks to the last advances in research concerning the molecular self-assembly process^1–4^. It is a natural process driven by various non-covalent interactions, such as electrostatic and/or hydrophobic, aromatic stacking, hydrogen bonding, or metal coordination interactions^5^. Generally, SAPs consist of monomers of short (8–16-mer peptides) or repeated sequences able to assemble to form well-ordered nanostructures which depend on amino acid sequence, the peptide concentration, pH, temperature, and the ionic composition of the medium^1^.

Peptide-based structures, compared to small synthetic molecules, possess several advantages like high chemical and biological diversity, higher potency and selectivity for their targets, low toxicity and good membrane penetration, and they are used in different fields^6,7^. Unfortunately, almost all peptides show poor metabolic stability and rapid clearance, making them not always suitable as drugs^8^. However, the modification of the SAPs’ chemical structure allows for regulating their size and morphology (e.g., micro/nanospheres, micro/nanoparticles, etc.)^9,10^. By modifying SAPs composition, in terms of length of the sequence and amino acid composition, it is possible to generate SAPs able to form structures with greater selectivity and stability than traditional non-biological materials. To reduce unwanted side effects, it is also possible to design peptides able to self-assemble into a particular biological target.

During the last few years, our group has successfully developed SAPs with many different functionalizations^1,11^, capable of boosting nervous regeneration. They also developed electrospun SAP microchannels entirely made of SAPs cross-linked with Genipin^12,13^, a natural-derived cross-linker already used in clinical trials, allowing the conjoint usage of the two new formulations of the SAPs scaffolds (hydrogel and microchannels) that will likely lead to a breakthrough in Spinal Cord Injury (SCI) treatments. In this work, we have analyzed the potentially toxic effects of SAPs and electrospun SAP made of natural amino acids that aim to be used in nervous tissue engineering (see Table 1). These SAPs (HS, 46) have been functionalized with active moieties interacting with cells and proteins, showed promising in vitro performances with neural stem cells^11,14,15^ and were successfully crosslinked^16^. Others (68, 65L), complementary SAPs^17^, are used during electrospun channel production as insulating layers preventing channel adhesion to the target where the scaffold is collected^18^. One (B42) is the best performing hydrogel of BMHP1-derived SAPs in vitro with neural stem cells^19^. Lastly, one (C1) showed great promise for high-performing biomechanics follwed by chemical cross-linking^20,21^. These SAPs additionally provide nanostructured microenvironments morphologically resembling the natural extracellular matrices (ECM).

**Table 1:** List of SAPs synthesized and used in this work.

Peptide	Sequence	Net charge	Type of SAP	Ref
46	FAQRVPPGGG(LDLK)_3_-CONH_2_	+ 2	LDLK12-based SAP	^11^
C1	Ac-CGG(LKLK)_3_GGC-CONH_2_	+ 5,9	Co-assembling SAPs family	^16,17^
B42	Biotin-GGGAFASAKA-CONH_2_	+ 1	BMHP1-derived SAP	^19^
HYDROSAP (HS)	Ac-SSLSVNDGGG(LDLK)_3_-CONH_2_ Ac-(LDLK)_3_-CONH_2_ Ac-KLPGWGGGG(LDLK)_3_-CONH_2_ [Ac-(LDLK)_3_G]_2_-KG(LDLK)_3_-CONH_2_	− 1 0 + 1 0	LDLK12-based SAPs comprising branched and linear SAPs developed for optimal 3D in vitro model of densely cultured hNSCs	^14^
65L	Ac-(LDLD)_5_-CONH_2_	− 10	Co-assembling SAPs family	^17^
68	Ac-FAQRVPPGGG(LDLD)_3_-CONH_2_	− 5	Co-assembling SAPs family	^17^

Net charges are calculated for pH = 7.

However, medical device use is subject to several laws, regulations, strict standards, and certification processes. Therefore, the development and manufacturing of medical devices have to consider the macroeconomic framework with specific factors regarding their production and use. Hence, translating SAPs into commercial products requires testing their harmless to animal and human tissues. In such a way that in this work, we have performed a multiparametric nanosafety profile of 6 different SAPs in vitro and in vivo using different official guidances developed to test the safety and medical devices’ biocompatibility.

## Results

### In vitro genotoxicity tests: micronucleus (MN) assay and TK gene mutation assay (GMA)

DNA reactive substances may directly damage DNA even when present at low concentrations, leading to mutations and potentially initiating cancer. Genotoxicity is a term that refers to the ability to interact with DNA and/or the cellular apparatus that regulates the fidelity of the genome, and mutagenicity refers to the induction of permanent transmissible changes in the sequence of the genetic material of cells or organisms. These mutations can involve a single gene or a block of genes.

Genotoxicity assessment is essential for developing medicines and ensuring the safety of industrial chemicals. In vitro assessment of genotoxicity precedes in vivo evaluation during drug development. The OECD has provided guidelines for the testing of chemicals using various in vitro genotoxicity tests, including the micronucleus test and the TK assay in the TK6 lymphoblastoid cell line (TG-487; TG-490).

**Figure 1:**
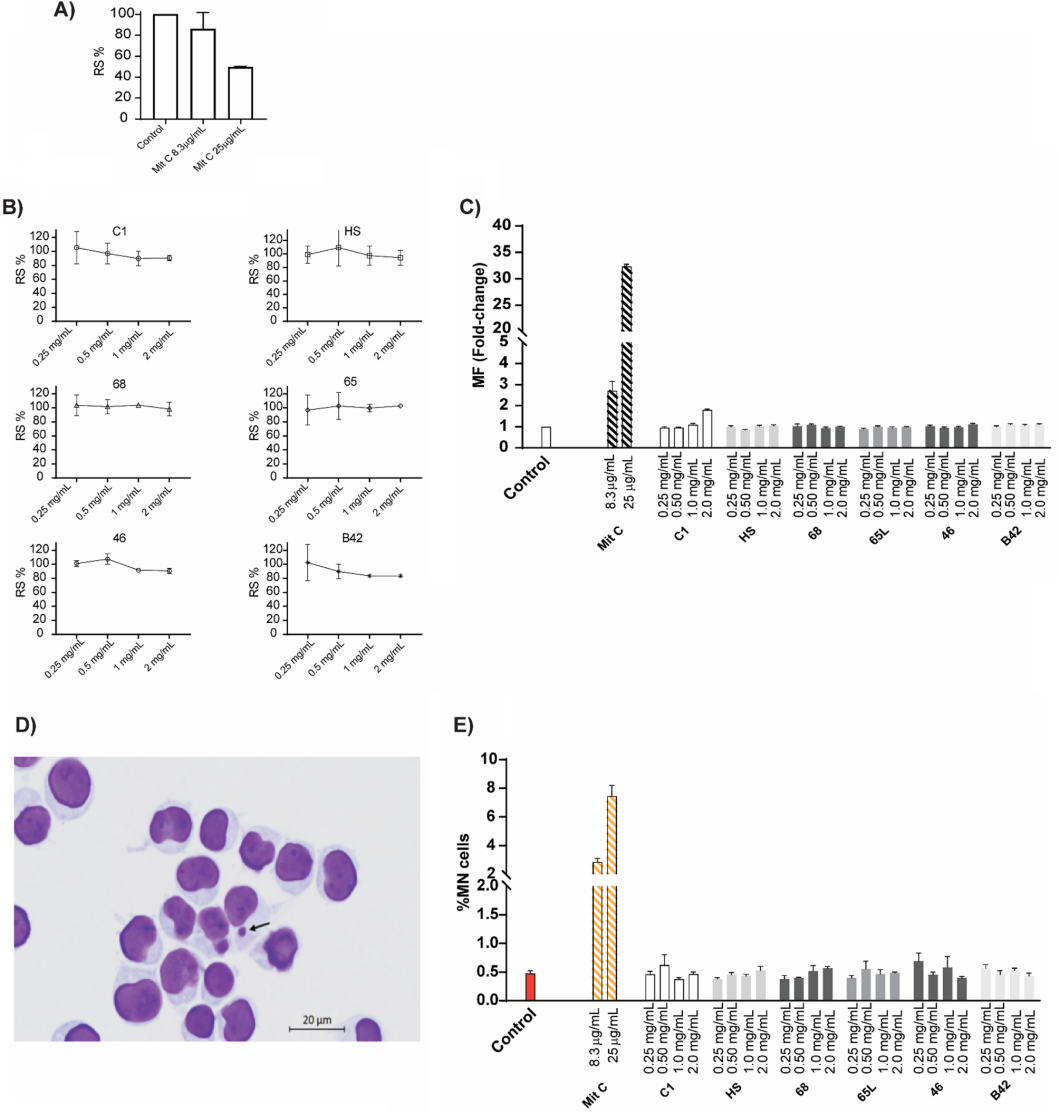
In vitro genotoxicity tests: the thymidine kinase (TK) gene and the micronucleus test assay in the TK6 human lymphoblastoid cell line. (**A**,**B**) Relative survival (RS) induced by SAPs was calculated by comparing the cloning efficiency (CE) of each SAPs with CE of the negative control determined after cultivating for 7–10 days and using the Poisson distribution method. Mitomycin C (MitC) was used as a positive control in A. (**C**) MF in TK-6 cells after 4 h of exposure to negative, positive controls and SAPs. (**D**) Photomicrographs of the cells scored in the MN assay. Arrow: a micronucleus in a mononucleated cell. (**E**) Percentage of micronucleated cells for each SAPs, mitomycin C (as positive control) and medium (as negative control). Data were expressed by mean ± standard deviation of the mean from three independent experiments. Statistical analyses were performed by one-way ANOVA. No significant changes regarding the control were detected.

To determine whether SAPs induce genotoxicity, TK6 cells were incubated with increasing concentrations of each SAP and positive and negative control (Mit C and culture medium, respectively). Cells were analyzed for CE via the recommended Poisson distribution method. As shown in Fig. 1A and B, in contrast to the positive control Mit C, cytotoxicity (RS) was not detected in any SAPs at any of the tested concentrations, even as high as reaching the mg/ml range. The RS% induced by SAPs was calculated with the ratio between the CE of each SAP molecule and the CE of the negative control (Fig. 1A,B).

For TK6 cells, the reported spontaneous mutant frequency (MF) is generally between 1–10 × 10^–6^. In this assay, the negative control MF was 5.4 × 10^–6^ while the positive controls (Mit C 8.3 and 25 µg/mL) were 14.6 and 175.3 × 10^–6^, respectively. Changes in MF relative to the negative controls (fold-change) were calculated and expressed graphically in Fig. 1C. None SAPs showed an increase in MF regarding negative control.

Furthermore, the MN was performed. The slides from the control and treated cells were examined under the microscope 48 h after chemical exposure. A total of 2000 cells were analyzed for every culture (Fig. 1D). None SAPs showed a significant difference with negative control. Mit C was used as positive control and showed a significant increase in the percentage of micronucleated cells (Fig. 1E).

### In vitro skin sensitization test

Skin sensitization is a multi-faceted process described as an Adverse Outcome Pathway (AOP). One of its steps is Dendritic Cell (DCs) activation, which migrates to the lymph nodes and induces T-lymphocyte proliferation and activation. Different models in vitro have been used to determine the sensitization potential of chemicals. Surface markers CD54/ICAM-1 (adhesion molecule) and CD86 (costimulatory molecule) are up-regulated in the presence of sensitizers and are indicative of DC maturation/activation. The changes in CD54 and CD86 are detected by flow cytometry following staining with fluorochrome-tagged antibodies. Concurrent cytotoxicity assessment is carried out to assess whether the up-regulated expression of CD54 and CD86 occurs at sub-cytotoxic concentrations of tested chemicals. The surface markers’ RFI is determined with respect to the solvent/vehicle control used.

**Figure 2:**
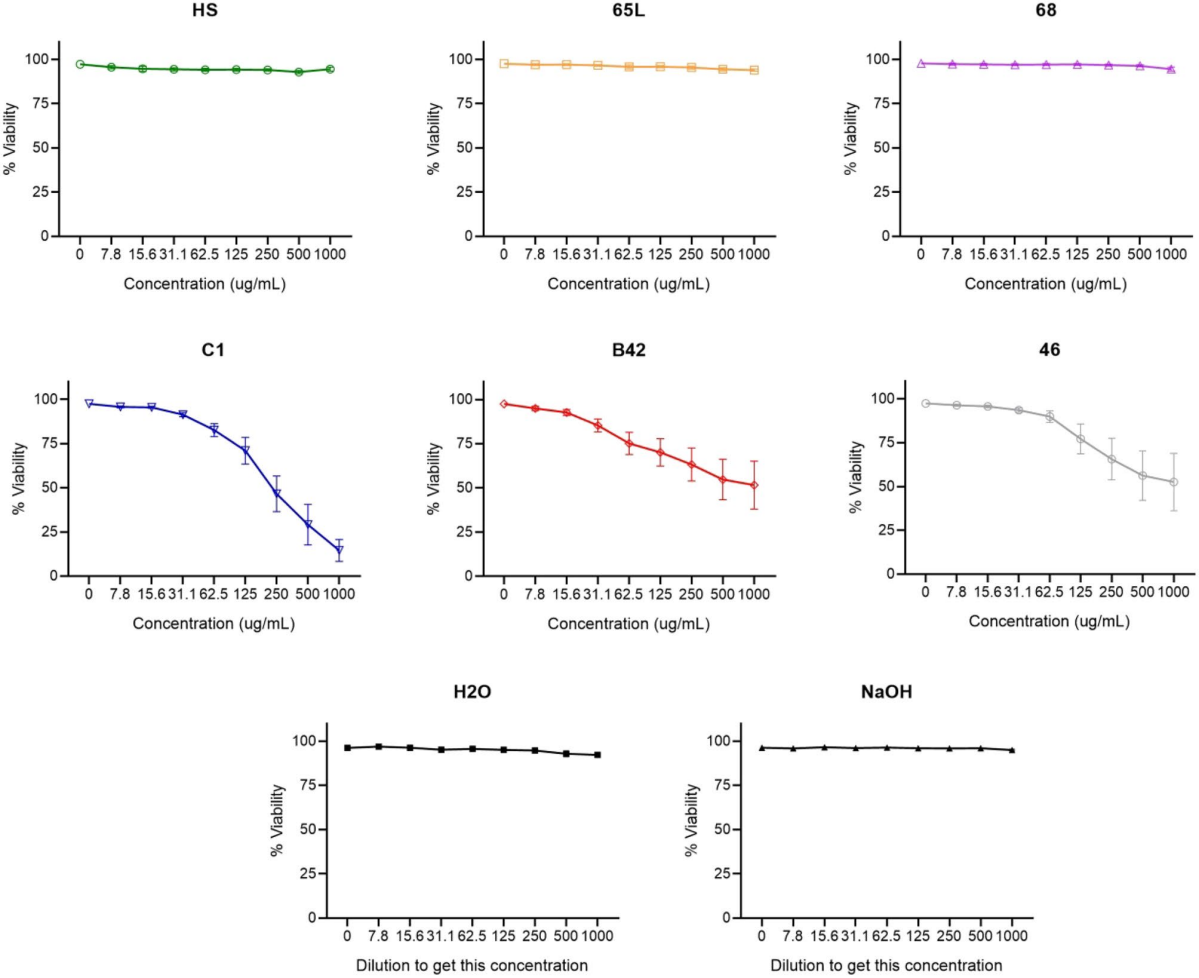
Effect of SAPs on THP1 cell death. THP1 cells (1 × 10^6^ cells) were treated with increasing concentrations of each SAPs for 24 h, as described in experimental procedures, and cell death was determined by measuring 7AAD staining and flow cytometry. Data correspond to the percentage of 7AAD negative cells. Data are expressed by mean ± standard deviation of the mean from three independent experiments.

The cells were incubated for 24 h with various concentrations of the SAPs and subjected to cell death staining with 7AAD. The results show that cell viabilities differed depending on SAPs. HS, 65L, and 68 showed no cytotoxicity in the concentration range tested. However, C1, B42, and 46 showed a different grade of cell death on THP1 cells after 24 h, decreasing cell viability proportional to the concentrations of the SAPs. Vehicles (H_2_O to HS, 46, C1, and B42, NaOH to 68 and 65L) were used as negative controls at the same dilutions used to prepare the SAPs, and the cell viability did not change during the 24-h incubation (Fig. 2). Concentrations over 1mg/mL could not be tested because the SAPs made cell culture medium gelatinous and viscous.

**Figure 3:**
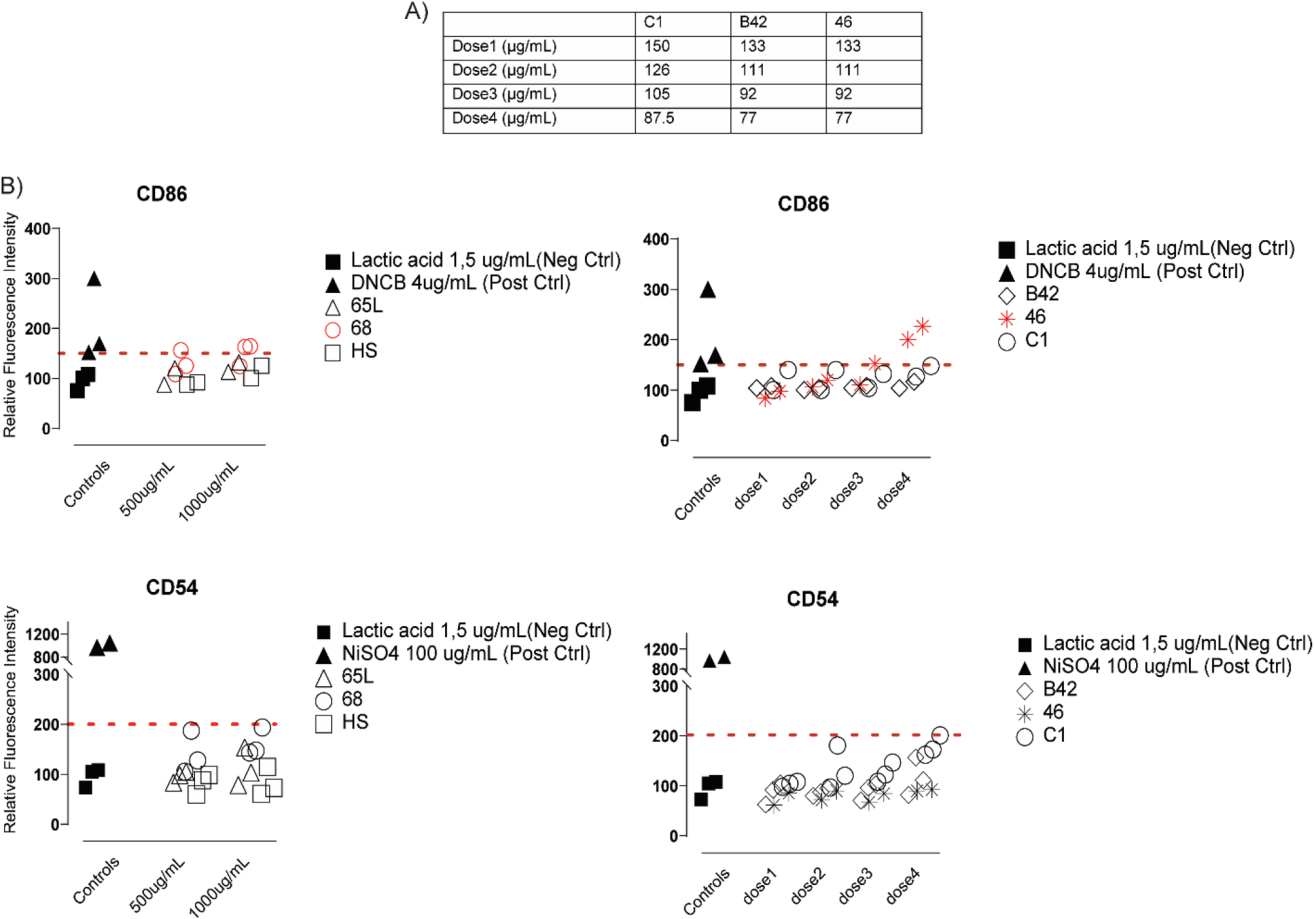
Skin Sensitization. Effect of SAPs on THP1 cell death. THP1 cells (1 × 10^6^ cells) were treated with increasing concentrations of SAPs for 24 h, and cell death was determined by measuring 7AAD staining and flow cytometry. (**A**) Concentrations selected to determine the expression of CD54 and CD86. The concentrations have been calculated from CV75 (concentrations that show 75% of THP-1 cell survival). (**B**) CD86, and CD54 (Relative Fluorescence Intensity (RFI) data for each SAPs, positive and negative control. In red are the sensitizer SAPs. The dots line indicates the maximum relative fluorescence Intensity threshold that can be considered negative results.

Then, the expression of CD86 and CD54 was analyzed by flow cytometry, and based on the geometric MFI, the RFI of CD86 and CD54 for positive, negative control cells and SAP-treated cells were calculated. The dose used to determine CD54 and CD86 expression of each SAP was calculated from that yielded 75% THP-1 cell viability (CV75) for C1, B42, and 46 (Fig. 3A). The doses used were the highest possible concentration for HS, 65L, and 68 SAPs (500 and 1000 µg/mL). After 24 h of incubation, variations of the RFI as an index of skin sensitization were determined. Figure 3B shows CD54 and CD86 expression for each of the six SAPs under investigation and negative and positive control. According to the results, 65L, HS, B42, and C1 SAPs can be classified as negative skin sensitizers. However, 46 and 68 SAPs are classified as positive skin sensitizers since two replicas give results over the threshold.

### In vivo evaluation of acute systemic toxicity

To address whether SAPs under study induce acute toxicity, rats received a subcutaneous injection (single dose) of the corresponding SAPs (10 mL/kg of body weight). The number of animals at the beginning of the study in each group and the initial and terminal mean of body weights and clinical adverse events observed after treatment are summarized in Table 2. Every animal included in the study (35 in total) completed the 5 days of the experiment. No toxicity signs during the evaluation of acute systemic toxicity were observed.

**Table 2:** Summarized data for each control and test group.

Group	References	Animals at the start of the test (n)	Adverse clinical signs (n)	Body weights at the beginning of the experiment (means)	Body weight at the end of the experiment (means)
HS	^14,15,16^	5	0	255.6 ± 8.25 g	261.8 ± 15.5 g
68	^17,18^	5	0	238.1 ± 8.05 g	247.9 ± 7.60 g
46	^11,16^	5	0	250.5 ± 9.10 g	265.2 ± 8.20 g
C1	^20,21^	5	0	238.8 ± 20.3 g	260.4 ± 19.5 g
65L	^17,18^	5	0	251.6 ± 19.7 g	271.8 ± 20.6 g
B42	^19^	5	0	243.8 ± 9.10 g	260.8 ± 10.9 g
Control		5	0	249.1 ± 16.1 g	264.1 ± 17.2 g

**Figure 4:**
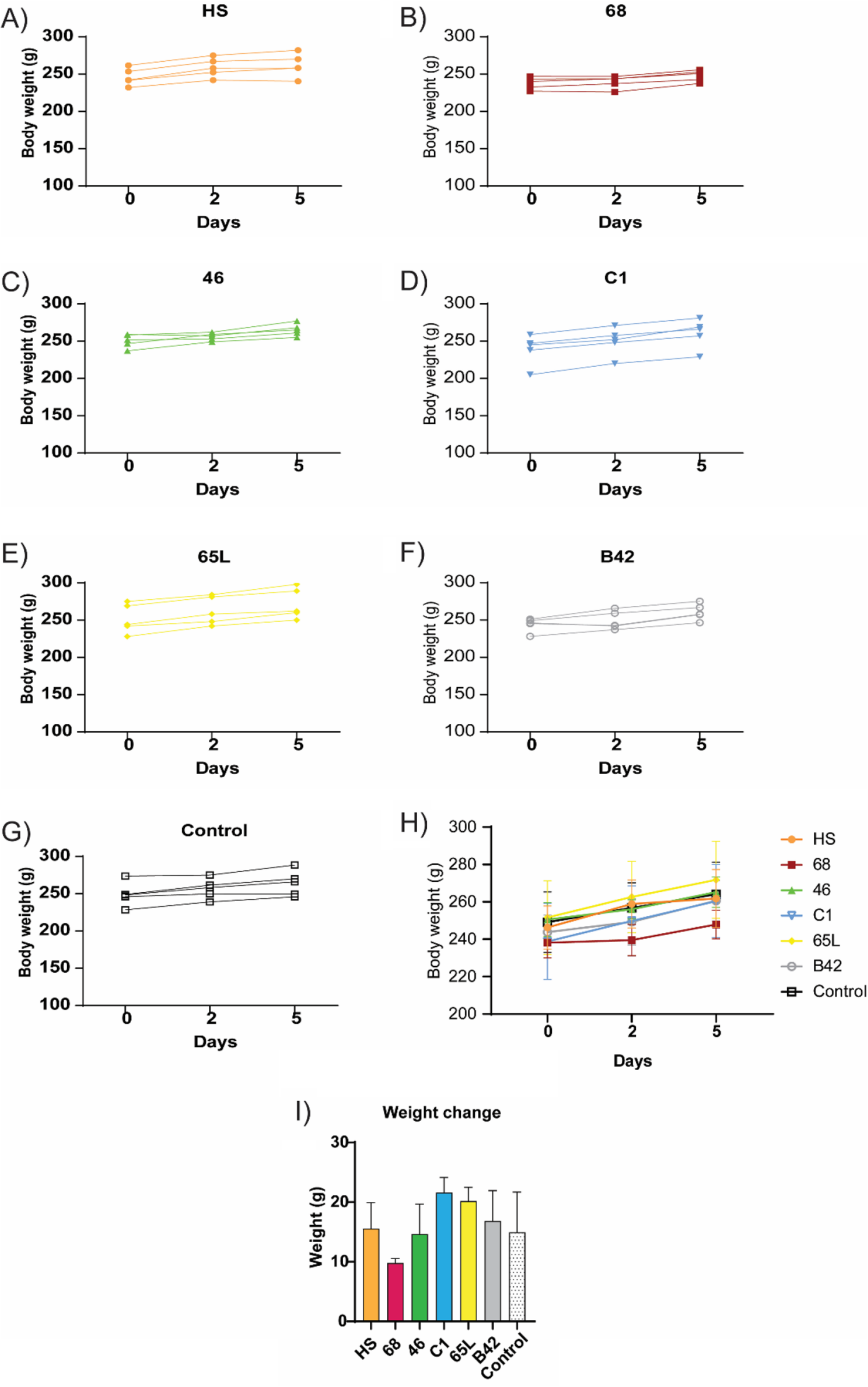
Acute systemic toxicity assay. (**A**–**G**) Body weight change of each animal at days 0, 2, and 5 (end of the study). (**H**) Mean body weight for each group at days 0, 2, and 5 (end of the study). (**I**) Weight increase from day 0 to day 5. Data are expressed by mean ± standard deviation of the mean. Rats were subcutaneously administered 10 mL/kg of body weight as described in the experimental procedure. Animals were weighed at days 0, 2, and 5. Statistical analyses were performed by two-way ANOVA test with Bonferroni post-hoc test. Animals were weighed at days 0, 2, and 5. No significant changes regarding the control group were detected.

Animals were weighed on days 0, 2, and 5 (Fig. 4, Table 2 and Supplementary Table 1). The body weight evolution of group 68 was significantly different from the control group (p = 0.03). However, although the group of SAP 68 had no weight increase on day 2, no significant changes were observed between groups, nor with the control group for each time point. At the study end, every animal increased their body weight.

**Figure 5:**
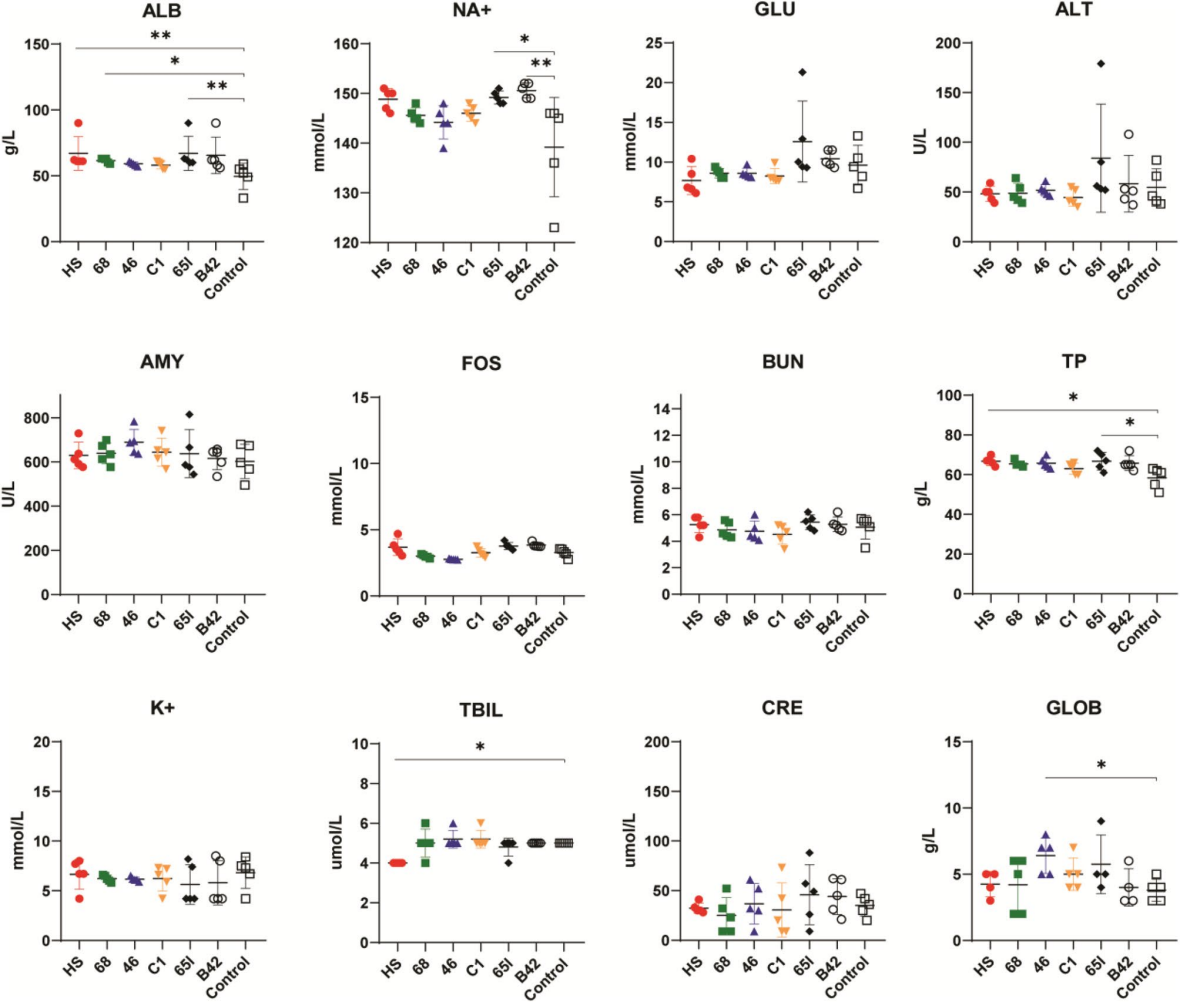
Evaluation of Biochemical parameters. ALB, Albumin; GLU, Glucose; ALT, Alanine Transaminase; AMY, Amylase; NA+, Sodium; BUN, Blood Urea Nitrogen; K, Potassium; FOS, Phosphorus; CRE, Creatinine; GLOB, Globulin; TBIL, Total Bilirubin; TP, Total Protein. Data are expressed by mean ± standard deviation of the mean. Statistical analyses were performed by one-way ANOVA. *p < 0.05; ** p < 0.01.

At the end of the study, the animals were sacrificed. Blood samples were obtained by cardiac puncture, and a dissection of every organ was performed. Blood sample biochemistry was analyzed with VetScan V2; the results are shown in Fig. 5. Significant higher albumin (ALB) levels in groups HS, 68, and 65L were observed compared with the control group; however, all the ALB value means, including the control group, were slightly above the reference; this is probably not associated with product toxicity. Sodium blood test (NA+) was also increased in the 65L and 68 groups, globulin blood tests (GLOB) in the group treated with SAP 46, total bilirubin (TBIL) in the HS group and total protein test (TP) in HS and 65L SAP groups. However, all of them were within the expected reference values (Supplementary Table 2). The other biochemical profiles of the tested animals did not differ significantly from control animals.

**Figure 6:**
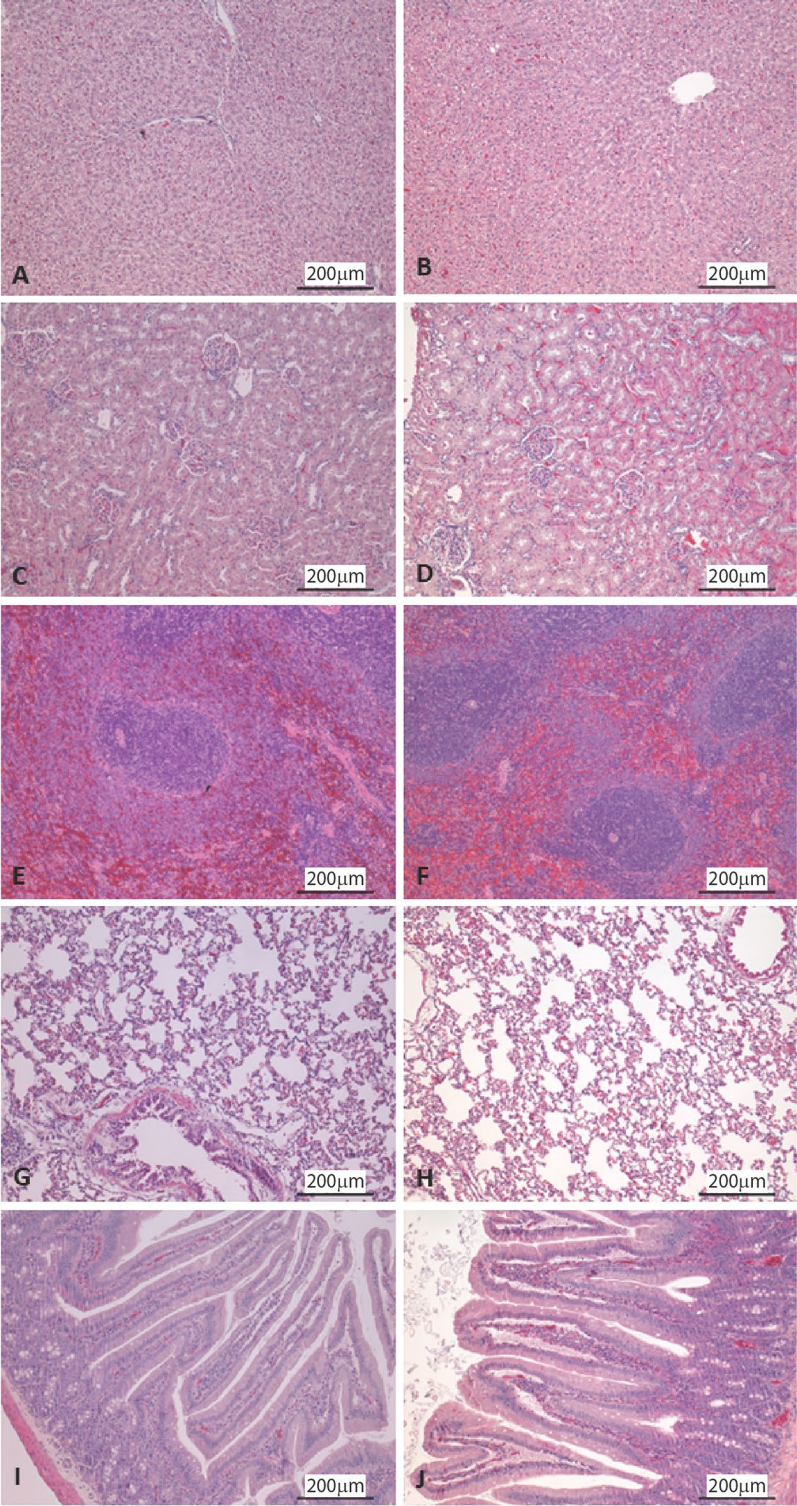
Histopathologic evaluation of animal organs after stained with hematoxylin–eosin. Histological images (10x) of control animals, (**A**) liver, (**C**) kidney, (**E**) spleen, (**G**) lung, and (**I**) duodenum; and treated animals (**B**) liver, (**D**) kidney, (**F**) spleen, (**H**) lung, and (**J**) duodenum.

No significant findings were observed on the macroscopic evaluation of the organs, including size, shape, color, and surface changes. The lungs, liver, spleen, kidneys, and duodenum were collected for histopathologic evaluation. After that, samples were processed and stained with H&E. No significant findings were observed in the samples from tested substances compared with tissues from the control group (Fig. 6). The liver showed a mild multifocal random inflammatory infiltrate. However, as the liver of the control animals presented the same image, it can be discarded as an effect of the drug administration. Due to the blood sampling method at the end of the study (cardiac puncture), some artifacts can be observed in the liver and lung.

We recently showed how microchannels made of cross-linked 46 and HS can be reproducibly produced and be satisfactorily seeded with human neural stem cells to obtain densely populated ATMPs^18^. Being hNSCs already GMP certified and used in approved clinical trials GMP for ALS^22^, we was prioritized the evaluation of the scaffold alone.

**Figure 7:**
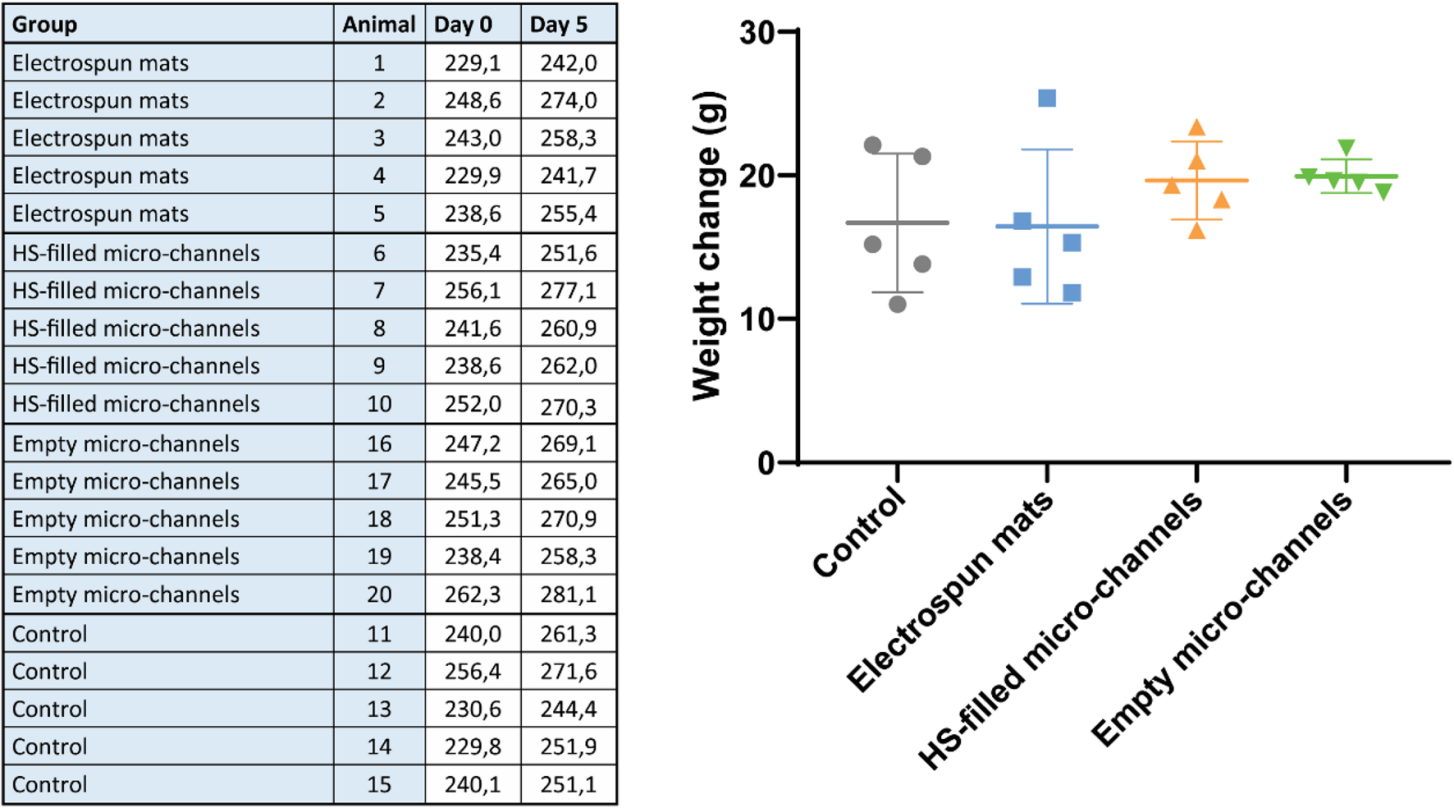
Medical devices acute biological evaluation. Body weight change of each animal after the medical device is subcutaneously implanted. Clinical signs of toxicity were not observed. Data are expressed by mean ± standard deviation of the mean.

**Figure 8:**
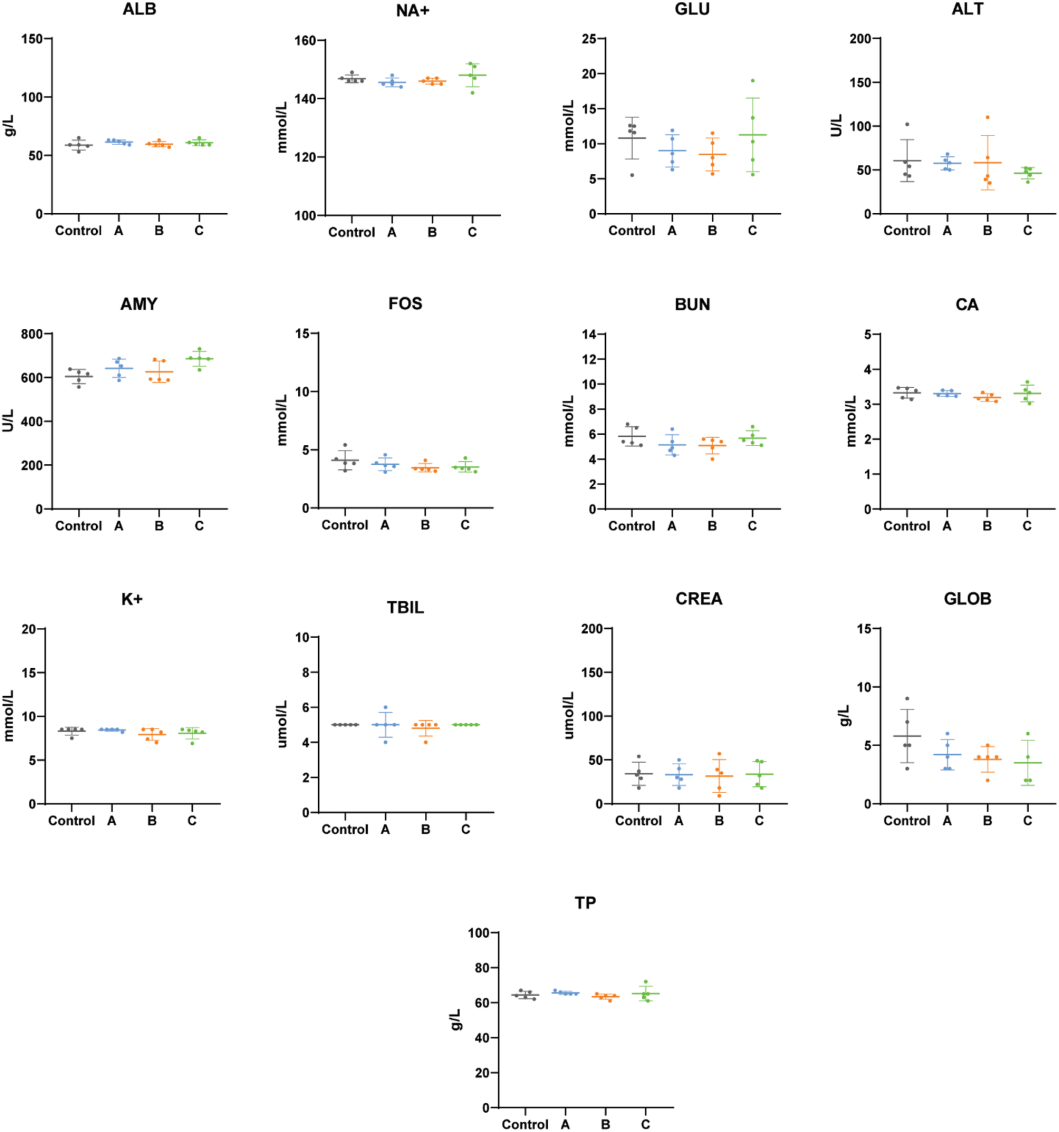
Biochemical parameters after acute exposition to medical devices. (**A**) Electrospun mats, (**B**) HS-filled micro-channels, and (**C**) empty micro-channels. ALB, Albumin; GLU, Glucose; ALT, Alanine Transaminase; AMY, Amylase; NA+, Sodium; BUN, Blood Urea Nitrogen; CA, Calcium; K, Potassium; FOS, Phosphorus; CRE, Creatinine; GLOB, Globulin; TBIL, Total Bilirubin; TP, Total Protein. Data are expressed by mean ± standard deviation of the mean. Data are expressed by mean ± standard deviation of the mean. Statistical analyses were performed by one-way ANOVA.

Similar results were observed in the medical device acute evaluation in vivo with the electrospun mats, empty micro-channels and HS-filled micro-channels, as shown in Figs. 7 and 8. All the animals completed the study without showing clinical signs of toxicity (Fig. 7). The biochemical profile of animals from each group showed no significant difference with control animals (Fig. 8). After histological evaluation of the lung, liver, spleen and kidneys, the result was the same as the SAPs, and we can presume that there is no systemic toxicity produced by the products tested (data not shown).

**Figure 9:**
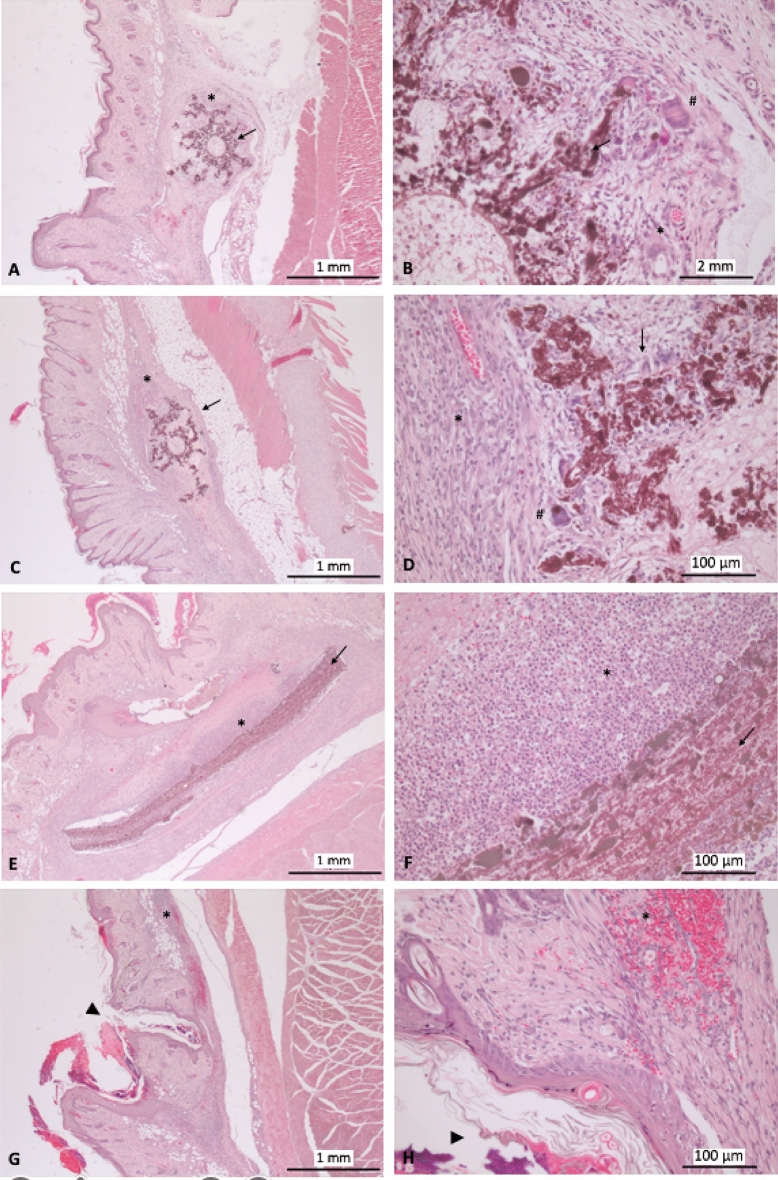
Histological evaluation of implant placement. (**A**,**B**) empty micro-channels. (**C**,**D**) HS-filled micro-channels. (**E**,**F**) Electrospun mats, and (**G**,**H**) control. implant (Arrow), Inflammatory infiltrate (*), multinucleated giant cell (#), and wound healing with keratine accumulation, normal process (arrowhead).

The skin where devices were implanted was also histopathologically evaluated. Implants (black implant in Fig. 9) were located in the dermal layer of the skin and appeared surrounded by an inflammatory response composed mainly of neutrophils and lymphoplasmacytic cells. Macrophages and multinucleated giant cells are also observed. Some of them absorb tiny beads of this black material. Besides, mild swelling and erythrocyte extra vascularization can be seen in the adjacent connective tissue. In some areas where this material appears ramified or fragmented in smaller pieces, body response tries to isolate them with connective tissue. This reaction is intense in group electrospun mats, probably due to the bigger size of the implant and mild-moderate in micro-channels, with no qualitative differences between them. Some connective tissue matrices and cells can be observed inside the tubes on the transversal section of materials. The inflammatory response is manifested as a foreign body reaction on the dermal layer. Based on the analyzed images, we hypothesize that the body could absorb them in more prolonged exposure (Fig. 9).

### SAPs increase the migration of endothelial, macrophage, fibroblasts and neuronal-like cells

How SAPs affect cell migration is an essential issue in a product that aims to improve tissue regeneration. Endothelial, macrophage, fibroblasts, and neuronal-like cells were drawn on to analyze how these SAPs modify cell migration. The monocytic cell line THP-1, the endothelioma cell linebEnd.3 cells, Mouse Embryonic Fibroblasts (MEFs) cells and the neuroblastoma cell line SH-SY5Y were used. THP-1 cells were differentiated into macrophages for this assay.

**Figure 10:**
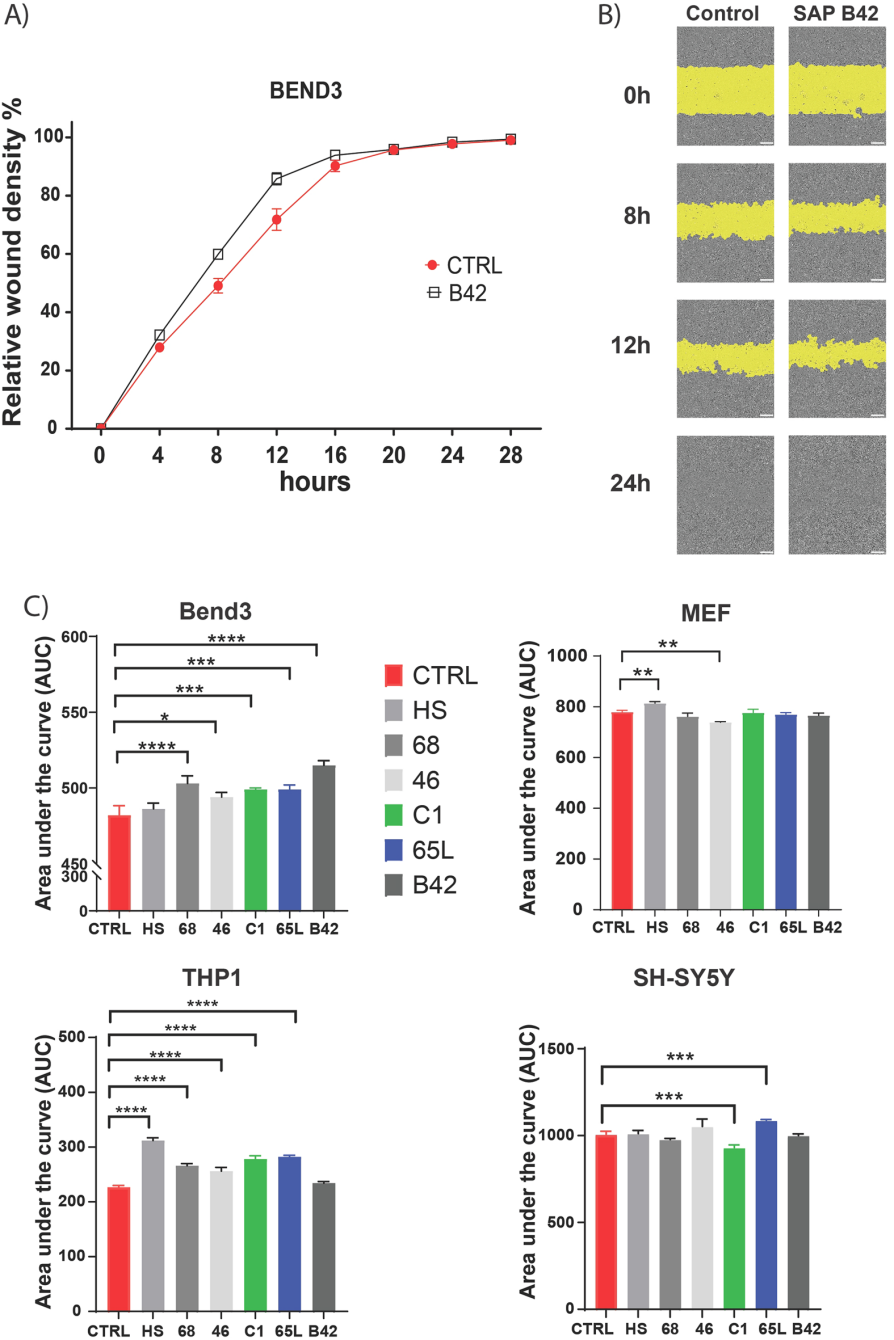
Wound healing assay to evaluate the effect of SAPs on cell migration. Representative graphics of relative wound density percentage vs time (**A**) and images at ×10 magnification. (**B**) from in vitro scratch wound healing assays. Cell migration into the cell-free region (in yellow) is accelerated in the presence of SAPs compared to controls. (**C**) Measurements of the area under the relative wound density percentage curve vs time for each SAP and cell line. Data are expressed by mean ± standard deviation of the mean from three independent experiments. White line 200 µm. Statistical analyses were performed by one-way ANOVA (****P < 0.0001, ***P < 0.001, **P < 0.01, *P < 0.05 versus control).

The wound healing assay is a standard in vitro technique for probing cell migration^23^. The basic steps involve making a linear thin scratch "wound" in a confluent cell monolayer and capturing images at the beginning and at regular intervals during cell migration to close the wound. This method involved measurements of how rapidly the initially-vacant area becomes re-colonized with cells as a function of time^24^. IncuCyte was used for this quantifying. The percent of relative wound density was plotted in the time to calculate the area under and compare each SAPs against the control. As can be observed, most of the SAPs increase the migration of these cells compared with the control, and only SAP C1 in SH-SY5Y and SAP 46 in MEF reduced cell migration (Fig. 10).

## Discussion

SAPs feature intrinsically prone to forming nanostructured solid substrates depending on their sequence. They have mainly been used as in vitro 3D systems as soft fillers or blended with stiffer synthetic polymers. However, they also match the biomimetics requirements that most tissue engineering approaches require SAP designs can incorporate specific features for individual tissues of interest, or a combination of features to accommodate specific needs, depending on their sequence. Thus, they are promising nanobiomateriales to be used in a wide range of biomedical applications including tissue repair and wound healing. Here we provide a very complete multiparametric characterization in vitro and in vivo of the nanosafety profile and potential toxic effects of different SAPs that have been previously developed as promising agents to treat Spinal Cord injuries.

SAP materials can be designed to perfectly match any need for biomimetic tunability required for most tissue repair and tissue engineering. With advances in mechanical tunability and 3D spatial organization, designer SAPs have been used as soft fillers for tissue repair and accelerated tissue wound healing as cartilage injury repair^25^.

Other SAPs advantages are the ability to produce designs with very high purity and being regulated as medical devices in most markers, making their commercialization easier. However, market approval of medical devices is based on regulatory compliance with relevant laws to demonstrate safety and performance in human use by biosafety studies. Hence, in order to determine the potential for an unacceptable adverse reaction from the contact of the device or its component with the body, the OECD has provided guidelines and International Organization for Standardization (ISO) norms for testing chemicals in developing medicines and medical devices. For example, chemical substances and their impurity may directly damage DNA even when present at low concentrations, leading to mutations and potentially initiating cancer. Similarly, the sensitizing effect is observed with chemicals and medical devices, causing a delayed hypersensitivity reaction. Hence, following the above-mentioned guidelines, our overall aim was to determine the SAPs’ toxicity.

To this aim, we have evaluated in vitro and in vivo six SAPs, and three electrospun SAP (electrospun mats, empty micro-channels and HS-filled micro-channels) developed by our team. It is especially important to elucidate genotoxicity and sensitization at an early stage of pharmaceutical development because this can avoid eliminating promising candidates at advanced stages of development after investing a lot of time and money in them^26^.

Our results regarding genotoxicity using these two assays, the TK and MN assay, showed that none of the six SAPs are genotoxic at the concentration assayed (Fig. 1). The TK assay is able to detect various mutations that inactivate the intact TK allelic gene, including point mutations, long deletion, DNA recombination, and chromosome loss^27,28^. On the other hand, the MN assay is induced by direct DNA damage (clastogenicity) or by retardation of chromosome segregation (aneugenicity). The target molecules of aneugens are not DNA but components of the mitotic apparatus, such as spindle fibers, in which aneugens interrupt the polymerization or depolymerization of tubulins. Therefore, DNA per se is not damaged by aneugens. Hence, these two assays are essential components of a genotoxicity test battery to detect different forms of genotoxicity. However, we did not detect any sign of toxicity using these two methods, which discards a potential genotoxic activity of these SAPs^29,30^.

During the development of new pharmaceuticals, genotoxicity assessment is an essential part of non-clinical safety evaluation. In fact, chemically synthesized peptides are often evaluated for genotoxicity like small drug substances, unlike biotechnology-derived proteins (antibodies, hormones, etc.) that share similarities to endogenous molecules that would not pose any genotoxic liability^31,32^. Of special concern, it should be stressed the presence of organic linkers in the chemically synthesized peptides, the presence of non-natural amino acids, chemical moieties in synthetic peptides and peptide size. All of these can facilitate membrane permeability and, therefore, access to the cytoplasm or nucleus like small molecules being able to affect maybe not DNA per se but aneugens^31^.

Sensitizers are chemicals with an intrinsic ability to induce allergy. Our results showed that SAPs 46 and 68 were classified as sensitizers. Sensitization is a multi-faceted process in which, after DC activation, DC migrates to the lymph nodes and induces T-lymphocyte proliferation. It is a type IV hypersensitivity reaction to an exogenous chemical mediated by T-cell-related processes, and the potential exposure to sensitizers becomes a greater regulatory issue within the biomedical industry^33,34^. Hence, regulatory agencies ensure the safety of all chemical ingredients in their products to avoid these adverse events. Here we used the h-CLAT assay that quantifies the changes in cell surface marker expression on THP-1 cells, a monocytic leukemia cell line that mimics DCs. In this assay, SAPs 46 and 68 increased the marker expression level indicating that these two SAPs are able to activate DCs and induce a hypersensitivity reaction. However, it should be noted that this induction was observed at a low level and only with the higher SAPs concentration. In the same assay, we also detected that C1 reduced the cell viability at high concentrations, an issue not detected in the previous assay incubating C1 only for 4 h (Figs. 1 and 2)^35^.

Systemic toxicity is the potential adverse effect of the use of medical devices. Hence, to evaluate the SAPs systemic toxicity and confirm the result observed in vitro we performed in vivo assays with the six SAPs and three electrospun. The in vivo evaluation of medical devices is performed to determine any potential adverse biological response resulting from contact with the component materials of the device with the body. Systemic and organ-specific effects can result from the absorption, distribution and metabolism of leachates from the device or its materials to body parts without direct contact. The device materials should not, either directly via surface-bound chemicals or physical properties or through the release of their material constituents, produce adverse local or systemic effects; unless it can be determined that the benefits of using that material outweigh the associated risks with an adverse biological response^36^.

We performed acute systemic toxicity by subcutaneously administering SAPs and implanting three electrospun following the ISO 10993-11 “Biological evaluation of medical devices—Part 11:Tests for systemic toxicity”. After sample administration, observations were made of adverse clinical signs, body weight change, gross pathological findings, and deaths. We evaluated the animals daily and did not detect any local or systemic adverse events. The histological organs’ H&E evaluation was similar between control and treated animals, and the same results were observed in the blood biochemistry determination (Figs. 4, 5, 6, 7, 8, 9).

These SAPs have been designed and functionalized to provide nanostructured microenvironments morphologically resembling the natural extracellular matrices to promote tissue repair and wound healing. Therefore, we evaluated the SAP impact on cell migration in four cell lines representative of cell types involved in wound repair. It is a complex and dynamic process of replacing devitalized and missing cellular structures and tissue layers that involve numerous cell types^37^. For this reason, we selected cell lines representative of vascular endothelium (Bend3) macrophage (THP1 differentiated into macrophage), Neuron (SH-SY5Y), and Fibroblast (MEF). Almost all SAPs increased the macrophages and endothelial cell migration. However, only SAP HS increased the fibroblast cell migration, while SAP 46 reduced the fibroblast migration. In the SH-SY5Y cells, the migration was only increased by the SAP 65L, while the SAP C1 reduced the cell migration. The other SAPs did not modify cell migration.

Analysis of cell migration in vitro is a functional assay to quantify alterations in cell migratory capacity in response to experimental manipulations. Wound healing is a complex cellular process to restore structurally damaged tissue^24^. Successful wound healing depends on many diverse processes and cell types. Most of SAPs tested increased the migration of macrophage and endothelial cells. These two cell types are critical in wound healing. Macrophages have a central role in tissue repair and regeneration, displaying versatility and high plasticity. Macrophages promote the scavenging of debris, bacteria and pro-inflammatory cells while stimulating reparative processes to allow effective wound resolution^38,39^.

On the other hand, the endothelial cells also have a unique role. They make the growth and survival of newly formed tissue possible, as all tissues depend on a blood supply, which in turn depends on endothelial cells. Therefore, new vessels must be formed by extension from the wound edges^24,40^.

## Conclusions

Our in vitro and in vivo results indicate that the SAPs tested are non-toxic when implanted, even when a slight sensitizer effect was observed during in vitro assays in two SAPs, which nevertheless do not affect animal health. These analyses, which are fundamental to evaluating the toxic effects of SAPs for biomedical applications using in vitro and in vivo assays from regulatory guidances, suggest that these SAPs might be tested in clinical trials preserving a good safety profile. Based on our results, the next step should be the development of phase I trials to support the safety of these products, as all preclinical in vitro and in vivo tests, albeit important, might present different limitations.

## Materials and methods

### Cell lines, chemicals, and reagents

All cell lines used in the present study were certified as free for mycoplasma. TK6, THP-1 and SH-SY5Y cell lines were obtained from the German collection DSMZ while bEnd.3 was from ATCC. The monocytic cell line derived from an acute monocytic leukaemia THP-1 was maintained in RPMI 1640 medium while bEnd.3 cells (derived from a murine endothelioma), MEFs cells (Mouse Embryonic Fibroblasts), and SH-SY5Y (a subline of the neuroblastoma cell line SK-N-SH) were maintained in Dulbecco’s Modified Eagle’s Medium (DMEM) (Lonza). Each medium was supplemented with 10% (v/v) heat-inactivated fetal bovine serum (FBS), 100 U/mL penicillin, 0.1 mg/mL streptomycin, and 2 mM L-glutamine. The lymphoblastoid TK6 cells were maintained in RPMI 1640 medium (Lonza) supplemented with 10% (v/v) heat-inactivated horse serum (HIHS) (Sigma), 100 U/mL penicillin/0.1 mg/mL streptomycin (Sigma), 2 mM l-glutamine (Sigma), 200 µg/mL sodium pyruvate (Sigma). All the cell lines were cultivated in a humidified incubator at 37 °C in an atmosphere of 5% CO_2_. Spontaneous TK6 cells thymidine kinase (TK)^−/−^ mutants were purged from working cell stocks by a 2-day exposure to CHAT medium (RPMI containing 10 µM deoxycytidine, 200 µM hypoxanthine, 0.1 µM aminopterin, and 17.5 µM thymidine) and then transferred into HCT medium (CHAT without aminopterin) for 2 days. Humans or human tissue specimens were not involved in the present study.

All SAPs were produced at the Center for Nanomedicine and Tissue Engineering (CNTE), Italy. The synthesis was carried out by following the protocol described by Ciulla et al.^20^, through Fmoc-solid phase peptide synthesis (SPPS) by using an automated synthesizer (Liberty Blue, CEM Corp., Matthews, NC, Canada) on a 0.25 mmol scale, DIC (N,N′-Diisopropylcarbodiimide) 1 M in DMF as activator solution, and Oxyma 1 M in DMF as activator base solution. A 10% v/v of piperazine in 9:1 NMP/EtOH was used for the removal of Fmoc groups. *N*-terminal acetylation was performed using a 20% v/v solution of Ac_2_O in DMF. Peptides were then cleaved from the resin with a cleavage cocktail of 92.5% TFA (trifluoroacetic acid), 2.5% H_2_O, 2.5% DODt (3,6-dioxa-1,8-octanedithiol), 2.5% TIS (triisopropyl silane). The obtained crude peptides were then purified by a Waters (Waters 1525, Waters Corp., Milford, CT, USA) binary reversed-phase high-performance liquid chromatography (RP-HPLC) using a C18 Restek™ column (C18, 5 µm, 150 × 30 mm) (Supplementary Fig. 1). The structure, identity, and purity of final products were checked through single quadrupole mass detection (Waters LC–MS Alliance 3100, Waters Corp., Milford, CT, USA) using nebulizing nitrogen gas at 400 L/min and a temperature of 250 °C, cone voltage 60 V, capillary 3.5 kV, and cone flow 40 mL/min, or with a Waters ACQUITY UPLC system (Waters Corp., Milford, CT, USA) coupled with a Waters Xevo G2-XS QT of Mass Spectrometer in a positive mode using a temperature of 150 °C, cone voltage 20 V, capillary 3 kV, and cone flow 20 mL/min, and by using a XBridge column (C18, 2.5 µm, 2.1 × 50 mm, Waters). The sequences described in this work are: FAQRVPPGGG(LDLK)_3_-CONH_2_ (46), Ac-CGG(LKLK)_3_GGC-CONH_2_ (C1), Ac-FAQRVPPGGG(LDLD)_3_-CONH_2_ (65L) and Biotin-GGGAFASAKA-CONH_2_ (B42), Ac-FAQRVPPGGG(LDLD)_3_-CONH_2_ (68), and HYDROSAP (HS)^14^, constituted of a mixture of the linear peptides Ac-KLPGWSGGGG(LDLK)_3_-CONH_2_, Ac-SSLSVNDGGG(LDLK)_3_-CONH_2_, Ac-(LDLK)_3_-CONH_2_, and the branched [Ac(LDLK)_3_G]_2_-KG(LDLK)_3_-CONH_2_. HS, 46, C1, and B42 stock solutions were made in distilled sterile water (GIBCO), while 68 and 65 L were dissolved in a solution of sodium hydroxide (25 mM), sonicated for 30 min until complete dissolution, and incubated overnight (ON) at 4 °C.

Nickel Sulphate (NiSO_4_, Sigma-Aldrich, 656895), 2,4-dinitrochlorobenzene (DNCB; Sigma-Aldrich, 237329), and lactic acid (LA, Sigma-Aldrich, PHR1215) were purchased from Sigma-Aldrich and selected based upon the criteria detailed in the general guidance from Organisation for Economic Cooperation and Development (OECD). The fluorophore-conjugated antibodies CD86-PE and CD54-APC were obtained from Miltenyi Biotec and Fc Block from BD.

All reagents (sodium dodecyl sulfate (SDS), hydroxide sodium (NaOH)) and solvents (hexafluoroisopropanol (HFIP), trifluoroacetic acid (TFA), dimethyl formamide (DMF), hydrochloric acid (HCl) and ethanol (EtOH)) were purchased from Merck (Merck Millipore, Darmstadt, Germany), Sigma Aldrich (Sigma Aldrich Chemie GmbH, München, Germany) and VWR (Radnor, PA, USA) in highest quality commercially available and used as received. Genipin (> 99% purity) was purchased from ChemNorm (Wuhan, China). Fmoc-protected amino acids and Rink Amide resin were obtained from CEM (Matthews, NC, USA).

### Cross-linked SAPs hydrogel preparation

Cross-linked peptides, 46gp, and HYDROSAPgp powder were prepared by adding 33 mg of Genipin dissolved in 10 ml of water: ethanol (80:20 v/v) to the 100 mg or 50 mg purified 46 or HYDROSAP powders, respectively, to achieve a final 1.33% or 0.83% w/v concentration solutions. It is worth underlining that Genipin had to dissolve quietly in the ethanol initially, and distilled water was then added gently to prevent Genipin precipitation. Afterward, the SAPs mixed solution was sonicated for 30 min and incubated at 37 °C for 72 h. Finally, the SAPs cross-linked solution with the dark blue color was flash frozen in liquid nitrogen and then lyophilized at − 50 °C for 72 h in a freeze dryer to achieve cross-linked peptides powder.

### Electrospinning of cross-linked SAPs scaffolds

Electrospinning solution for SAPs-based fibers was prepared by dissolving 37wt% of 46:HYDROSAP:SDS (89:10:01) in a mixture of solvents containing HFIP and TFA (99:1 v/v) (Table 1). The 46, HYDROSAP, and SDS were dissolved in the HFIP:TFA by continuously vortexing to form a homogeneous solution. Subsequently, the solution was sonicated for 30min at room temperature (RT) (bath temperature kept low by a glass of ice), and afterward, the solution was used straight away for electrospinning. The fibers were electrospun using Electroris (FNM Ltd., http://www.fnm.ir) as an electrospinning device having precise humidity and temperature controller. Briefly, the solutions were loaded in a syringe (diameter d = 4.6 mm, BD Micro-Fine) placed in the horizontal direction. The positive electrode was connected to the needle 29G (diameter d = 0.33mm) of the syringe and the negative electrode was attached to the collectors. A circular flat collector covered with an aluminum sheet (diameter 7.5 cm) and rotating 33G needles mounted as a target in the rotating arm of the mandrel are used to fabricate 2D fibrous lamina and 3D fibrous microchannels, respectively. The distance between the tip and the collector was set to 80 mm, and the scanning range and rate were adjusted to 50mm and 1000 mm/min to uniformly distribute electrospun fibers on the collectors. A controllable syringe pump in the range of 0.01–100 ml/h was used to feed the needle. The applied parameters were: voltage tension = 17/18 kV, tip-collector distance = 8 cm, flow rate = 20–40 µl/h, humidity = 45% and temperature = 22 °C.

The electrospun mats obtained were dried by a vacuum drier at RT for one hour before post-treatment to allow for trapped solvents to evaporate.

Electrospun mats and microchannels obtained were annealed by vapor exposure to the 1–3 ml of 25 mM solution of NaOH in H_2_O for 3 days at 37 °C. After the annealing, insoluble mats were dipped in a solution of Genepin 4% in EtOH:PBS (20:80 v/v) for 1 day at 37 °C. In this way, the Genepin could accomplish to crosslink the self-assembled peptides, increasing their stability. Finally, the post-treated mats could be stored at 4 °C in PBS.

### In vitro genotoxicity tests: micronucleus (MN) assay and thymidine kinase gene mutation assay (TK GMA)

The general guidance from OECD TG 487 and 490 were followed^26,41^. TK6 cell suspensions were treated with serially diluted SAPs molecules at 37 °C for 4 h, washed and resuspended in fresh medium, and cultured in new flasks for the MN and TK assays. We conducted the MN test and the TK mutation assay 48 h and 72 h later, respectively.

To measure cytotoxicity in the assay (i.e., relative survival [RS]), cells were distributed in flat-bottom 96-well plates (Nunc^®^, Thermo Fisher Scientific) at 1.6 cells/well in 10% (v/v) HIHS growth medium. These cells were cultivated for 7–10 days and then analyzed for cloning efficiency (CE) via the recommended Poisson distribution method.

To measure genotoxicity, cells were subcultured at a maximum density of 2.5 × 10^5^ cells/mL (at most 20 × 0^6^ cells) for 3 additional days to allow phenotypic expression of the TK gene prior to mutant selection. Forty-eight hours after treatment, the cells were prepared for the MN assay. Briefly, a small portion of cell culture from the flask was transferred to a 15 mL tube and centrifuged at 1200 rpm for 5 min at RT. The supernatant was then removed, and the pelleted cells were resuspended in PBS and placed in a cytospin funnel where cells were cytospun for 4 min at 900 rpm on microscope coverslips. A Diff-Quik staining was performed for microscope scoring. At least 2000 intact cells for each treatment were examined, and the cells containing MN were scored. Cells containing one or more micronuclei were recorded as micronucleated cells. Micronucleated cells should have the following characteristic: the micronuclei have a diameter < 1/3 of the diameter of the nucleus, the micronuclear boundary is distinguishable from the nuclear boundary (no overlapping), cells with a well-preserved cytoplasm, and mononucleated cells. The MN frequencies between non-treated and treated cells and the concentration–response relationship were statistically analyzed. As a positive control, mitomycin C (Mit C) was used.

The cultures were maintained for another 24 h to allow phenotypic expression prior to plating for TK assay to determine the mutant fractions. Briefly, the cells were distributed in 96-well plates at 4 × 10^4^ cells/well in a selection medium that contained 4 μg/mL trifluorothymidine (TFT) (Sigma) to kill those cells carrying an intact TK gene. Appearing *TK* mutants were enumerated by eye (under magnification) following cultivation for 14 days. Fresh TFT selection medium was then reapplied to the cells before any late-appearing TK mutants were enumerated 7–10 days later. Mutation frequencies (MFs) were finally calculated from cells grown in TFT selection medium or non-selection medium (for 7 days) via the Poisson distribution method. Test chemicals that induced concentration-dependent increases in MFs compared to control were genotoxic in this version of the TK GMA. Changes in MFs relative to the negative controls (fold-change) were calculated and expressed graphically.

### In vitro skin sensitization test

To perform in vitro skin sensitization test guidance OECD 442E was followed^42^. In the dose-finding assay (determination of CV75 values), THP-1 cells were seeded at 0.2 × 10^6^ cells/ml and cultured for 24 h. Before starting the assay and after the initial seeding time, substances were solubilized in a complete RPMI cell culture medium. Cells were then seeded into 96-well plates and dosed with over an 8-dose range. Solvent and culture medium were used as the negative control. After 24 h, the cells were centrifuged and washed twice with 200 μl PBS. Cells were then suspended in 200 μl containing 7AAD and analyzed with a MACSQuant10 flow cytometer (Miltenyi). The percentage of cell viability was determined and used to calculate the CV75 (concentration at which 75% of the cells are alive). At least three independent experiments were done.

To perform CD54 (ICAM-1) and CD86 expression measurement, THP-1 cells were seeded into 24-well plates at 1 × 10^6^ cells/well, dosed with SAPs as well as positive (2,4-dinitrochlorobenzene and Nickel Sulphate) and negative (lactic acid and medium) controls, and analyzed for cell surface marker expression with a MACSQuant10 flow cytometer (Miltenyi). CD86-PE and CD54-APC were used as fluorophore-conjugated antibodies. In line with the requirements stipulated in OECD TG442E, doses were based on the CV75 value, with the highest dose being 1.2 × CV75. NiSO_4_ (100 μg/ml) and DNCB (4 μg/ml) were used as positive controls, and lactic acid (1.5 μg/ml) as a negative control. After 24 h, the cells were centrifuged and washed twice with 200 μl staining buffer. Cells were then suspended in a blocking buffer containing Fc Block and incubated at 4 °C for 15 min. After blocking, supernatants were removed, and the conjugated antibodies were added to each cell pellet as required. After thorough pipette mixing, plates were incubated for 30 min at 4 °C. After incubation, the cells were centrifuged and washed three times with 200 μl staining buffer. Cells were then suspended in 200 μl FACS buffer containing a yellow live/death probe and analyzed on the flow cytometer. The geometric mean fluorescence intensity (MFI) from each sample was determined and used to calculate the Relative Fluorescence Intensity (RFI), which was then used in the prediction model described in the OECD TG442E. The RFI was calculated as shown below by the expression levels of CD86 and CD54.

$${\text{RFI}}\left(\mathrm{\%}\right)=\frac{\mathrm{MFI \; of \;cells \;with \;SAPs }- \mathrm{MFI \; of \; isotype \; control \;cells }}{\mathrm{MFI \;of \; vehicle \;control \;cells}-\mathrm{MFI \; of \;isotype \;control}} \times 100$$

The prediction model used in this method based on CD86/CD54 expression measurement stipulates that a tested chemical is considered POSITIVE if at least one of the following conditions is met in 2 of 2 or at least in 2 of 3 independent runs; otherwise, the h-CLAT prediction is considered NEGATIVE.

- The RFI of CD86 is equal to or greater than 150% in at least one tested concentration (with cell viability ≥ 50%);
- The RFI of CD54 is equal to or greater than 200% in at least one tested concentration (with cell viability ≥ 50%).

### Scratch wound assay

Prior to initiating the assay, cells were grown to confluence in a 96-well plate at 37 °C in an atmosphere of 5% CO_2_ and, with the help of a 96-pin IncuCyte^®^ Wound Maker simultaneously created precise and reproducible wounds in all wells of a 96-well plate by gently removing the cells from the confluent monolayer. After wounding, immediately aspirate the media from each well and wash the cells twice with culture media (100 µL/well). After washing, 100 µL of media containing 0.01 mg/mL of the appropriate agent was added to the corresponding well, and the plate was placed inside the IncuCyte. Assay plates were then equilibrated within the IncuCyte^®^ for a minimum of 15 min before the first scan. The software was set to scan the plate every 4 h for migration assays.

### In vivo tests for systemic toxicity

The general guidance from the ISO10993 to Biological evaluation of medical devices was followed. Animals were housed in plastic cages with free access to drinking water and a pelleted basal diet under controlled conditions of temperature (22 °C ± 3 °C), humidity (50 ± 10%) and light (12/12 h light/dark cycle). Rats were quarantined for the first 7 days and randomly divided into experimental and control groups. All animals were bred and maintained at CIBA (Biomedical Research Center of Aragon; Department of Experimental Surgery). Animal experimentation was approved by the Ethics Committee for Animal Experimentation from the University of Zaragoza (protocol number PI31/22), and all authors complied with the ARRIVE guidelines.SAPs or electrospun mats, empty micro-channels, and HS-filled micro-channels were subcutaneously administered to female Sprague–Dawley rats (*Rattus norvegicus*) 7 weeks old, non-pregnant, and nulliparous. The concentration of each SAP was: HS: 1%, 68: 3%, 46: 5%, C1: 3%, 65L: 3% and B42: 1%. On the day before administration, the animals were weighed, and the hair of their thoracic back was shaved. On day 1, the different SAPs were administered in one single dose by subcutaneous injection to each rat corresponding to each group. To devices, a skin incision of about 1 cm and subcutaneous dissection were performed under sterile conditions. Then, the products provided were introduced into the subcutaneous space, one piece per animal. Animals were weighed. Animals were euthanized by trained personnel using medical-grade Carbon dioxide (CO_2_). CO_2_ inhalation is the most common method of euthanasia used for small animals (i.e., mice, rats, guinea pigs, and hamsters). It is considered an acceptable euthanasia agent for small animals when properly administered. It was accepted by the Ethics Committee for Animal Experimentation from the University of Zaragoza (protocol number PI31/22). For histological assessment, lung, liver, spleen, kidneys, and duodenum were removed from rats, fixed with 4% paraformaldehyde, and embedded in paraffin. Sections from these samples were stained with hematoxylin and eosin (H&E). Blood samples were drawn at the end of the study by cardiac puncture. They were analysed with VetScan V2.

### Statistical analyses

Data are expressed by mean ± standard deviation of the mean. Body weight comparison between groups was assessed using the repeated measures two-way ANOVA test. Bonferroni posthoc multiple comparisons were also used. Analysis of biochemical parameters was made with one-way ANOVA. They were performed using GraphPad Prism 8 (8.0.2).

### Ethics approval and consent to participate

Animal experimentation was approved by the Ethics Committee for Animal Experimentation from the University of Zaragoza, protocol number PI31/22. Animals were euthanized by trained personnel using medical-grade Carbon dioxide (CO_2_). CO_2_ inhalation is the most common method of euthanasia used for small animals (i.e., mice, rats, guinea pigs, and hamsters). It is considered an acceptable euthanasia agent for small animals when properly administered. It was accepted by the Ethics Committee for Animal Experimentation from the University of Zaragoza (protocol number PI31/22). Humans or human tissue specimens were not involved in the present study.

### Supplementary Information

Supplementary Information.

## Data Availability

The datasets used and/or analysed during the current study are available from the corresponding author on reasonable request.

## Abbreviations

ALB: Albumin
AOP: Adverse Outcome Pathway
CE: Cloning efficiency
DC: Dendritic cell
DIC: N,N′-Diisopropylcarbodiimide
DMF: Dimethyl formamide
DNCB: 2,4-Dinitrochlorobenzene
DODt: 3,6-Dioxa-1,8-octanedithiol
ECM: Extracellular matrices
EtOH: Ethanol
H&E: Hematoxylin and eosin
HCl: Hydrochloric acid
HFIP: Hexafluoroisopropanol
LA: Lactic acid
MEFs: Mouse Embryonic Fibroblasts
MFI: Mean fluorescence intensity
MFs: Mutation frequency
Mit C: Mitomycin C
MN: Micronucleus
NaOH: Hydroxide sodium
NiSO_4_: Nickel Sulphate
OECD: Organisation for Economic Cooperation and Development
RFI: Relative Fluorescence Intensity
RS: Relative survival
RT: Room temperature
SAPs: Self-assembling peptides
SCI: Spinal Cord Injury
SDS: Sodium dodecyl sulfate
SPPS: Solid phase peptide synthesis
TFA: Trifluoroacetic acid
TFT: Trifluorothymidine
TIS: Triisopropyl silane
TK GMA: Thymidine kinase gene mutation assay
