# Supplementary material for: Multiparametric in vitro and in vivo analysis of the safety profile of self-assembling peptides
Source: Sci Rep. 2024 Feb 22;14:4395. doi: 10.1038/s41598-024-54051-7 (PMC10883997; doi:10.1038/s41598-024-54051-7)

**Supplementary**

Supplementary Table 1. Body weight

| Grupo | Animal | Día 0 | Día 2 | Día 5 |
| --- | --- | --- | --- | --- |
| HS | 1 | 242.0 | 258.0 | 258.3 |
| HS | 2 | 261.8 | 275.0 | 282.0 |
| HS | 3 | 241.8 | 252.4 | 258.2 |
| HS | 4 | 253.6 | 267.0 | 270.1 |
| HS | 5 | 232.1 | 241.9 | 240.4 |
| 68 | 6 | 232.8 | 237.2 | 242.7 |
| 68 | 7 | 247.0 | 246.9 | 255.8 |
| 68 | 8 | 227.2 | 226.1 | 237.4 |
| 68 | 9 | 240.0 | 243.9 | 250.7 |
| 68 | 10 | 243.5 | 243.7 | 252.8 |
| 46 | 11 | 246.7 | 259.0 | 265.0 |
| 46 | 12 | 237.0 | 249.0 | 255.0 |
| 46 | 13 | 258.3 | 262.0 | 277.0 |
| 46 | 14 | 259.0 | 257.0 | 268.0 |
| 46 | 15 | 251.7 | 253.0 | 261.0 |
| C1 | 16 | 238.0 | 248.2 | 257.0 |
| C1 | 17 | 247.0 | 257.8 | 266.0 |
| C1 | 18 | 205.0 | 220.0 | 229.0 |
| C1 | 19 | 245.0 | 252.0 | 269.0 |
| C1 | 20 | 258.9 | 271.0 | 281.0 |
| 65L | 21 | 228.0 | 242.0 | 250.0 |
| 65L | 22 | 244.0 | 258.0 | 262.0 |
| 65L | 23 | 269.0 | 281.0 | 289.0 |
| 65L | 24 | 242.0 | 248.0 | 260.0 |
| 65L | 25 | 275.0 | 284.0 | 298.0 |
| B42 | 26 | 249.0 | 259.0 | 266.7 |
| B42 | 27 | 228.0 | 237.0 | 246.4 |
| B42 | 28 | 246.0 | 242.0 | 257.2 |
| B42 | 29 | 245.0 | 242.7 | 257.8 |
| B42 | 30 | 251.0 | 265.7 | 275.1 |
| Control | 31 | 248.4 | 258.2 | 266.1 |
| Control | 32 | 246.0 | 249.9 | 249.6 |
| Control | 33 | 249.0 | 261.5 | 270.1 |
| Control | 34 | 273.7 | 275.1 | 288.6 |
| Control | 35 | 228.4 | 239.1 | 245.9 |

Supplementary Table 2. References value of biochemical profiles in rats.

|  | **Ref. Values** |
| --- | --- |
| ALB (g/L) | 38-48 |
| ALT (U/L) | 35-80 |
| TBIL (µmol/L) | 3-9 |
| BUN (mmol/L) | 3.6-7.5 |
| Ca (mmol/L) | 2-3.25 |
| Fos (mmol/L) | 1.71-2.68 |
| CREA (µmol/L) | 44-88 |
| Na (mmol/L) | 140-150 |
| K (mmol/L) | 4.3-5.6 |
| TP (g/L) | 56-76 |

**Supplementary Figure 1**. Mass analysis to each peptide. The structure, identity, and purity of final products checked through single quadrupole mass detection.


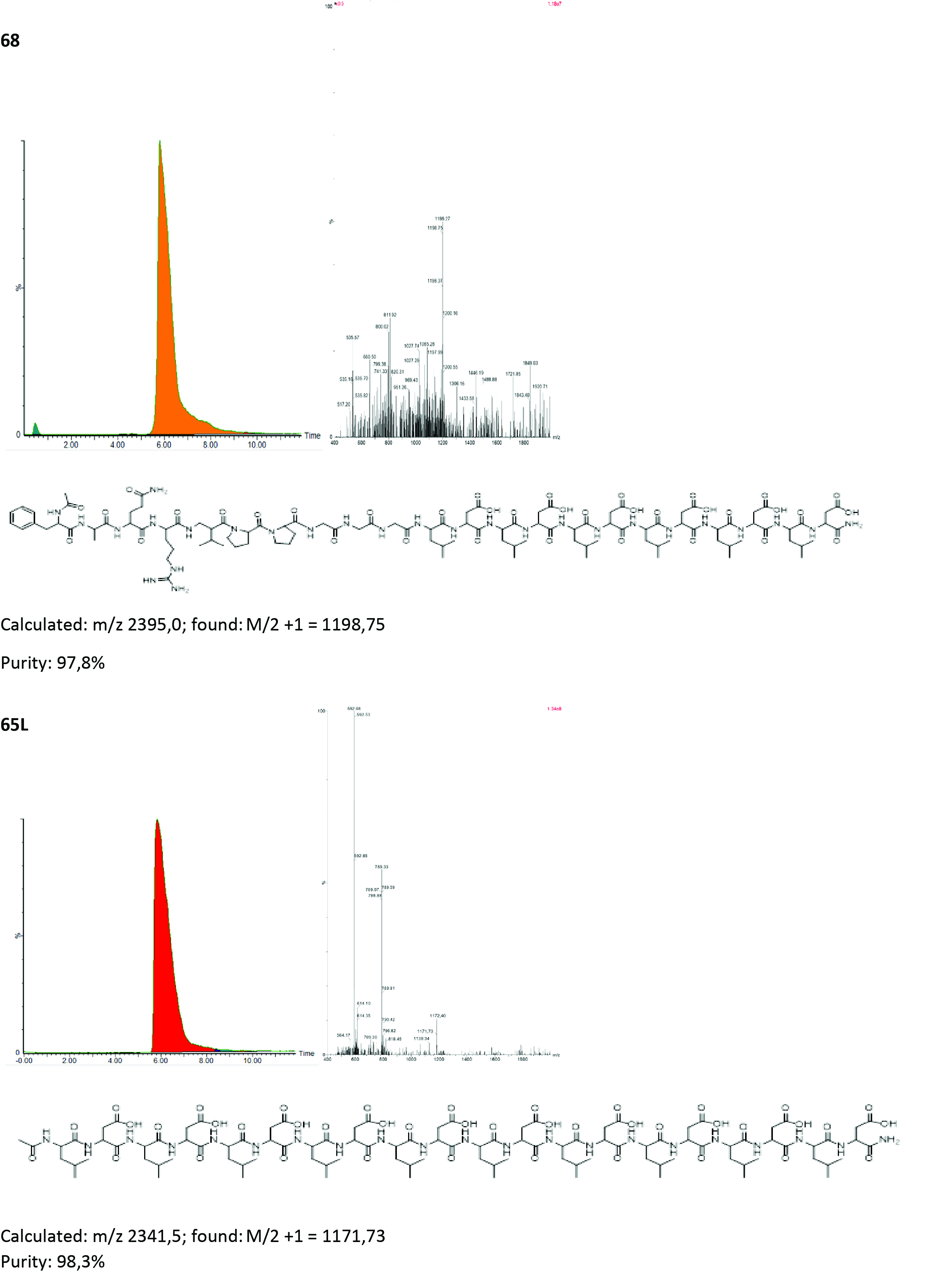


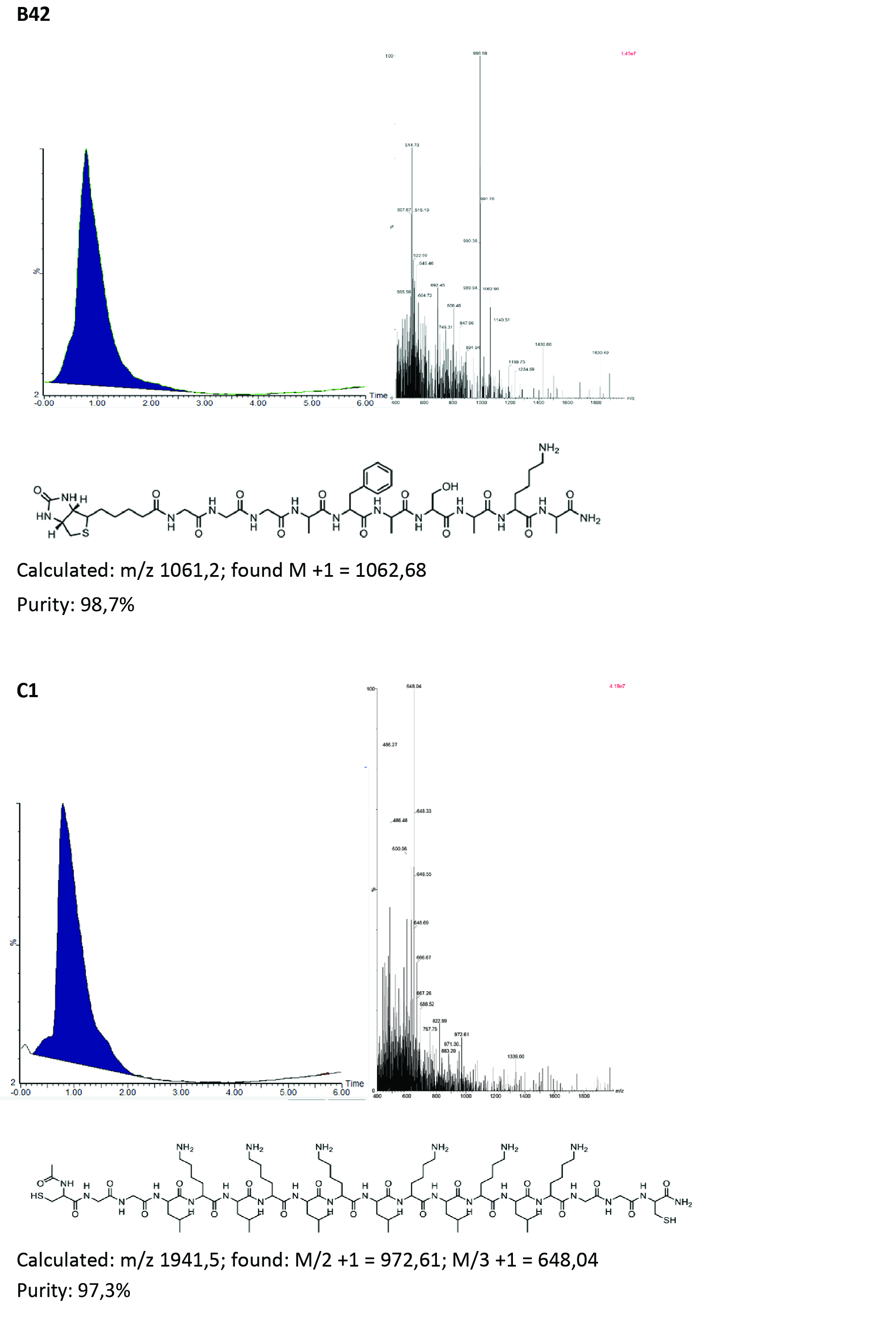


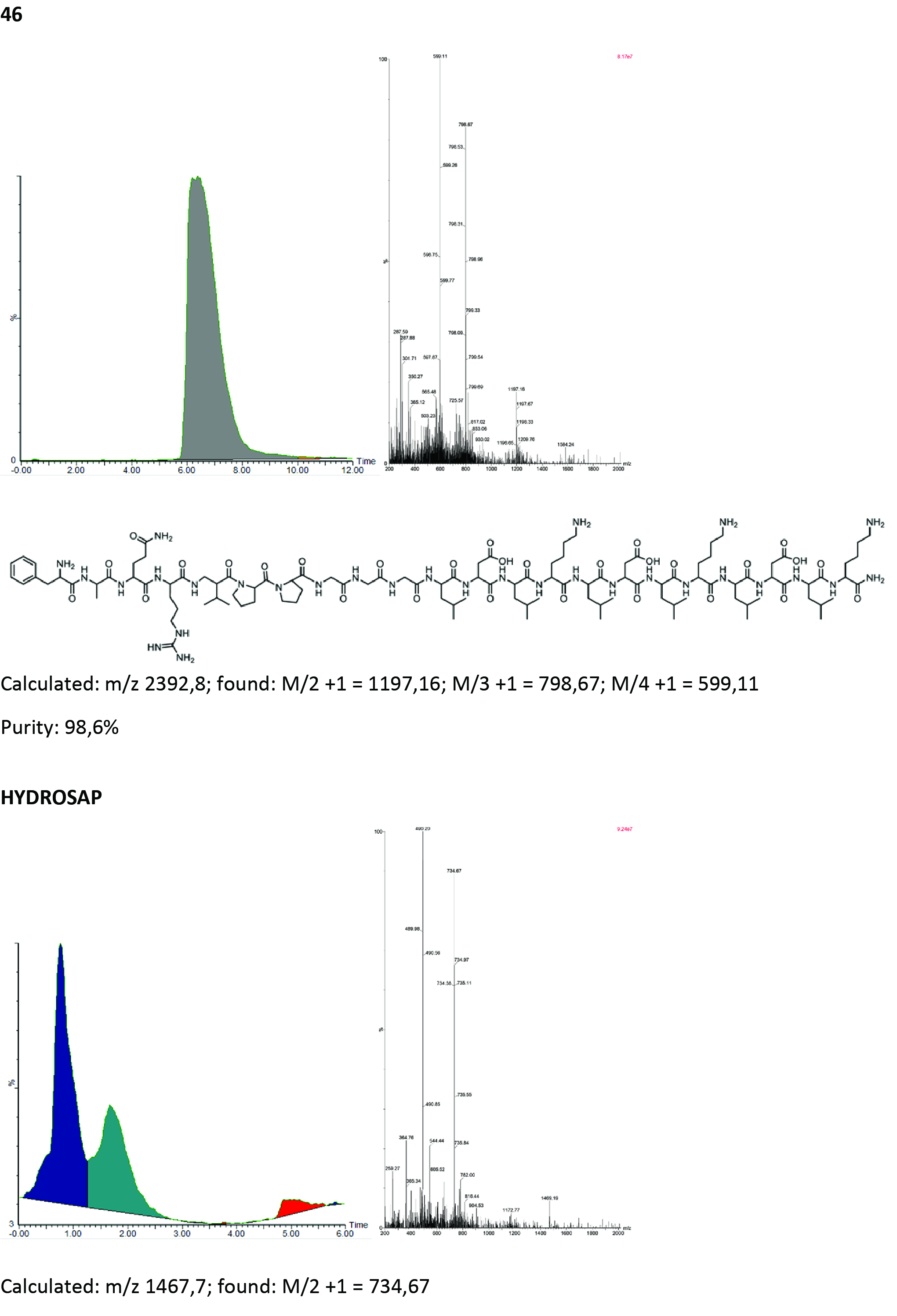

Supplement: Supplementary file 1 — Supplementary Information. [file 41598_2024_54051_MOESM1_ESM.doc]
